# Genome sequencing-based transcriptomic analysis reveals novel genes in *Peucedanum praeruptorum*

**DOI:** 10.1186/s12863-023-01157-y

**Published:** 2023-09-18

**Authors:** Cheng Song, Yingyu Zhang, Yunpeng Zhang, Shanyong Yi, Haoyu Pan, Ranran Liao, Yuanyuan Wang, Bangxing Han

**Affiliations:** 1 Anhui Dabieshan Academy of Traditional Chinese Medicine, Anhui Engineering Laboratory for Conservation and Sustainable Utilization of Traditional Chinese Medicine Resources, Anhui Engineering Research Center for Eco-agriculture of Traditional Chinese Medicine, College of Biological and Pharmaceutical Engineering, West Anhui University, Lu’an, 237012 China; 2 Henan Key Laboratory of Rare Diseases, The First Affiliated Hospital, College of Clinical Medicine of Henan, University of Science and Technology, Luoyang, 471003 China; 3 Shanghai Key Laboratory of Regulatory Biology, School of Life Science, East China Normal University, Shanghai, 200241 China; 4 School of Life Science, Anhui Agricultural University, Hefei, 230036 China; 5 School of Pharmacy, Anhui University of Chinese Medicine, Hefei, 230012 China

**Keywords:** *Peucedanum praeruptorum*, High-throughput sequencing, Coumarin, Lignification, Transcriptional regulation

## Abstract

**Background:**

*Peucedanum praeruptorum* Dunn, a traditional Chinese herbal medicine, contains coumarin and volatile oil components that have clinical application value. However, early bolting often occurs in the medicinal materials of Apiaceae plants. The rhizomes of the medicinal parts are gradually lignified after bolting, resulting in a sharp decrease in the content of coumarins. At present, the link between coumarin biosynthesis and early bolting in *P. praeruptorum* has not been elucidated.

**Results:**

Combining the genome sequencing and the previous transcriptome sequencing results, we reanalyzed the differential transcripts of *P. praeruptorum* before and after bolting. A total of 62,088 new transcripts were identified, of which 31,500 were unknown transcripts. Functional classification and annotation showed that many genes were involved in the regulation of transcription, defense response, and carbohydrate metabolic processes. The main domains are the pentatricopeptide repeat, protein kinase, RNA recognition motif, leucine-rich repeat, and ankyrin repeat domains, indicating their pivotal roles in protein modification and signal transduction. Gene structure analysis showed that skipped exon (SE) was the most dominant alternative splicing, followed by the alternative 3’ splice site (A3SS) and the alternative 5’ splice site (A5SS). Functional enrichment of differentially expressed genes showed that these differentially expressed genes mainly include transmembrane transporters, channel proteins, DNA-binding proteins, polysaccharide-binding proteins, *etc*. In addition, genes involved in peroxisome, hexose phosphate pathway, phosphatidylinositol signaling system, and inositol phosphate metabolism pathway were greatly enriched. A protein-protein interaction network analysis discoverd 1,457 pairs of proteins that interact with each other. The expression levels of six *UbiA* genes, three *UGT* genes, and four *OMT* genes were higher during the bolting stage. This observation suggests their potential involvement in the catalytic processes of prenylation, glycosylation, and methylation of coumarins, respectively. A total of 100 peroxidase (*PRX)* genes were identified being involved in lignin polymerization, but only nine *PRX* genes were highly expressed at the bolting stage. It is worth noting that 73 autophagy-related genes (*ATGs*) were first identified from the KEGG pathway-enriched genes. Some *ATGs*, such as *BHQH00009837*, *BHQH00013830*, and *novel8944*, had higher expression levels after bolting.

**Conclusions:**

Comparative transcriptome analysis and large-scale genome screening provide guidance and new opinions for the identification of bolting-related genes in *P. praeruptorum*.

**Supplementary Information:**

The online version contains supplementary material available at 10.1186/s12863-023-01157-y.

## Background

*P. praeruptorum* is a perennial angiosperm belonging to the Peucedanum genus of the Apiaceae family. The only source of the traditional Chinese medicine coverd by the Chinese Pharmacopoeia is the dried root of *P. praeruptorum*. The flavor profile of *Peucedanum spp* is characterized by bitterness, pungency, and a subtle coolness. This therapeutic approach is commonly employed for the management of symptoms such as panting with phlegm heat, expectoration of yellow and thick phlegm, and wind-heat cough with excess phlegm. Additionally, it offers the advantages of phlegm resolution, wind expulsion, and heat clearance [1]. Coumarin compounds are the main active ingredients of *P. praeruptorum*, which exhibit anti-tumor property, vasodilatory effects, and hypotensive activity [2, 3]. Dihydropyranocoumarins are the main medicinal ingredients in *P. praeruptorum* [4]. Some key genes involved in the biosynthesis and transport of *P. praeruptorum* coumarins have been successfully identified using transcriptome and metabolome techniques. It was believed that CYP450 family genes and MDR transporters were involved in the biosynthesis and transport of coumarins [5, 6]. Many studies have proved that *PAL*, *4CL*, *C2’H*, *BMT*, *COMT*, *AS*, *PS*, etc. are the essential genes for forming coumarin [7–12]. Our study showed that the phenylpropane biosynthesis pathway, the ABC transporter, the genes involved in apoptosis, and the circadian rhythm genes are potentially significant in controlling signal transduction of bolting, as well as the biosynthesis and transport of coumarin [13].

In recent years, there has been a discrepancy between the supply of wild Peucedanum plants and the demand in the market [14]. *P. praeruptorum* seedlings enter vegetative growth after sowing and emergence in the first year, and the roots are thick and fleshy. It can be used as medicinal material when excavated in the autumn and winter of that year. Due to differences in genetic background and growth environment, the growth speed of individuals varies greatly, and some *P. praeruptorum* seedlings may bolt early and enter reproductive growth. Whether early bolting occurs or not has become a decisive factor affecting the quality and harvesting of *P. praeruptorum* [15].The scientific mechanism of the sharp reduction of coumarin content after bolting is not clear. The apical part becomes the growth center after bolting. The majority of the nutrients produced by photosynthesis are moved from the roots to the apical region, resulting in the roots not obtaining enough nutrients for maintaining regular metabolic activities. This has led to an increase in secondary xylem area and a decrease in coumarin content [16, 17].

Plants belonging to the genus *Peucedanum*, *Angelica*, *Saposhnikovia*, *Notopterygium*, and *Glehnia* have long been confronted with the issue of rendering their roots unsuitable for medicinal utilization subsequent to the onset of bolting and flowering. The phenomenon of early bolting significantly hampers the accumulation of secondary metabolites in traditional Chinese medicine, hence imposing significant constraints on the clinical application of medicinal resources [18]. Despite the presence of coumarin in many Apiaceae plants, there has been a relative scarcity of study conducted on the prenyltransferase (PT), *O*-methyltransferase (OMT), and UDP-glycosyltransferase (UGT) of *P. praeruptorum*. These enzymes play crucial roles in the biosynthesis of umbelliferone. The comprehensive investigation of the ABC transporter genes responsible for the transmembrane transport of coumarins remains limited. By collecting experimental samples at different harvest periods, different bolting times, and different slope directions, we carried out high-throughput sequencing and multi-omics analysis of *P. praeruptorum*. Among them, phenylpropane pathway genes, ABC transporters, apoptosis-related genes, and circadian rhythm regulation genes play important regulatory roles in regulating bolting and flowering, coumarin biosynthesis and transport [13]. In this study, we aligned the previous transcriptome sequencing results with the reference genome of *P. praeruptorum* and identified a series of new transcripts that differently expressed after bolting. In addition to the previously studied coumarin biosynthetic genes, including the *PT* family (a branch of *UbiA*), *UGT* family, and *OMT* family. A large number of *PRX* family genes were identified, suggesting that a few *PRX* genes may be involved in lignin polymerization in roots. More than 70 potential *ATGs* were isolated by the functional annotation of differentially expressed genes. Nearly one-third of *ATG* genes were highly expressed during bolting, which may explain the apoptotic and senescent processes in roots. The specific functions of these *ATG* family genes need further characterization.

## Results

### Quality control, genome alignment, and identification of novel transcripts

**Fig. 1:**
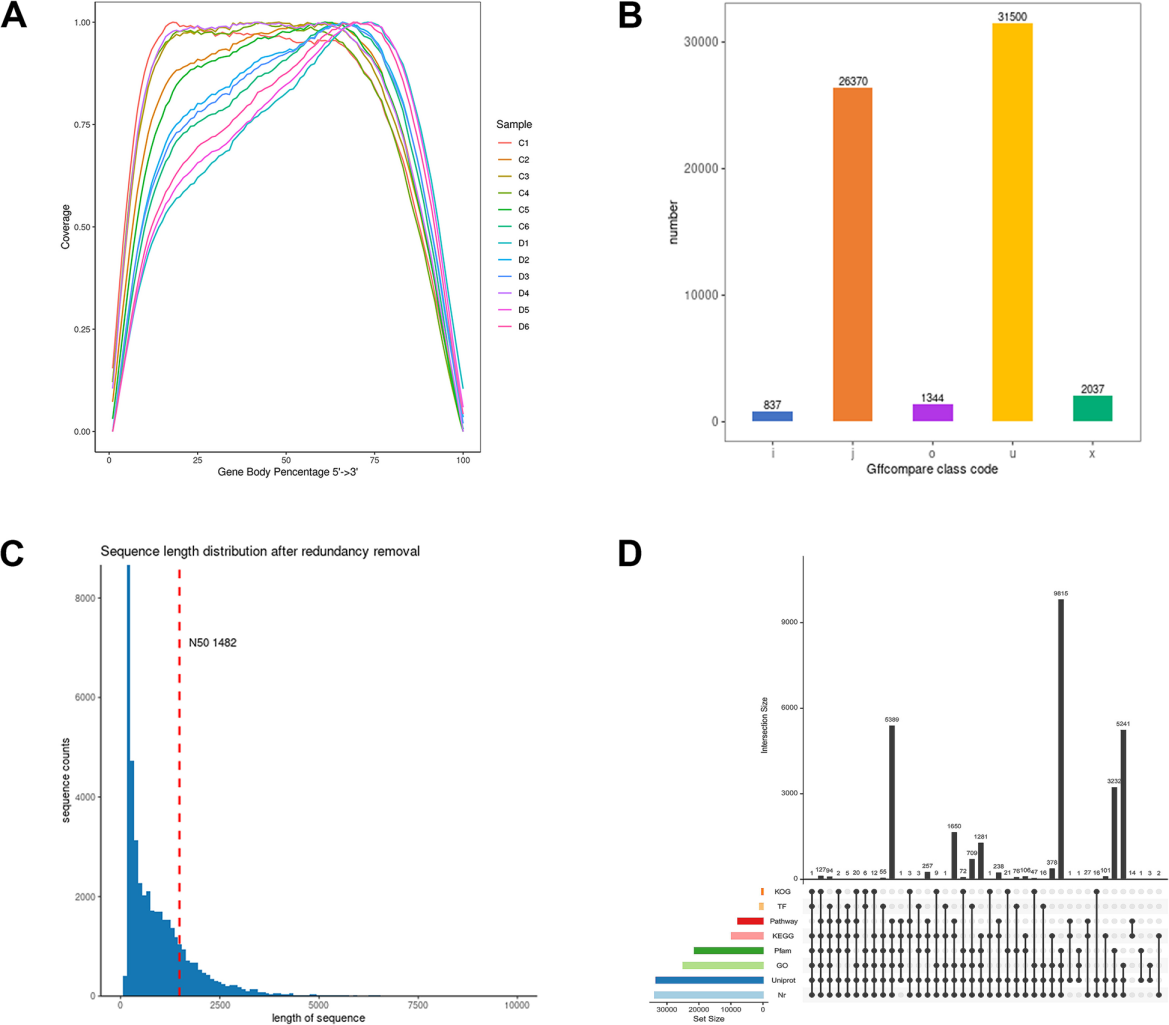
Alignment analysis of reference genomes and novel transcript annotations. **A** Random distribution of Reads on the reference gene. The abscissa is the relative position of the gene, and the ordinate is the proportion of reads in the total alignment in the corresponding position interval, and different colors represent different samples. This reflects the coverage of the gene (along 5’ to 3’) by the reads. **B** Quantity distribution of different types of new transcripts. o is the other part of the same strand overlapping with the reference exon; j is at least one matching multi-exon; x is the exon overlap on the anti-strand; i is the intron completely contained in the reference transcript Middle; u is an unknown new transcript. **C** CDS length distribution of new transcripts. The abscissa indicates the length of reads; the ordinate indicates the number of reads within the length range, and the red dotted line indicates the length of N50. **D** Distribution of the number of new transcripts annotated by different databases

 First, the raw data files acquired from the previous high-throughput sequencing were subjected to base calling analysis to convert them into original reads [5, 13]. Secondly, we removed low-quality and adapter sequences to obtain clean reads. After filtering, the Q20 and Q30 values of the clean reads of the 12 samples were all greater than 97% and 93%, indicating that the sequencing quality was excellent (Table 1). The GC content of these clean reads ranged from 42 to 45%. Subsequently, the filtered transcriptome sequences were aligned with reference genes. The unique mapped sequences of all samples accounted for more than 85% of the reference sequences, indicating that most of the transcripts could be matched to the reference genome (Table 2). The degree of randomness of mRNA fragmentation was assessed by conducting additional analysis on the position distribution of reads on the gene. The results show that there are noticeable variations in the coverage of reads that can be compared with the genome across different samples. The majority of the samples exhibited a favorable degree of matching, ranging from 10 to 75%. However, the relative position distribution proportion declined when it fell below 10% or exceed 75% (Fig. 1A). By comparing the transcripts of all the samples that combined with the known transcripts of the genome, it was indicated that the new transcript types in the samples mainly consisted of five distinct categories, of which 31,500 were unknown new transcripts and 26,370 were multi-exon transcripts. The remaining three categories had relatively low number of transcripts (Fig. 1B). The quantification of the novel transcripts of varying lengths was achieved by predicting the coding sequence (CDS) of these transcripts and determining the amount of reads associated with them. The length of N50 was 1,482 bp. As the length of the transcript increases, the number of reads it contains gradually decreases (Fig. 1C). Eight databases were employed to annotate the functions of these new transcripts, and a total of 54.60% of the transcripts could be annotated, among which the Nr database annotated the highest proportion, 54.38% (Table 3). The largest proportion of the 33,898 new transcripts were jointly annotated by the four databases Pfam, GO, Uniprot, and NR (Fig. 1D).

**Table 1:** Sequencing data obtained after filtering

Sample	Raw reads	Raw bases	Clean reads	Clean bases	Q20_rate	Q30_rate	GC_content
C1	39,209,858	5,881,478,700	37,466,596	5,290,294,198	0.97607	0.93329	0.43874
C2	50,740,794	7,611,119,100	48,999,980	6,977,576,588	0.97731	0.93472	0.43318
C3	42,979,798	6,446,969,700	41,552,716	5,909,944,266	0.9781	0.93635	0.43256
C4	42,391,360	6,358,704,000	40,438,006	5,720,213,564	0.9766	0.9349	0.43704
C5	45,187,404	6,778,110,600	43,348,392	6,093,787,668	0.97505	0.93048	0.44647
C6	32,546,920	4,882,038,000	31,169,300	4,331,795,722	0.97714	0.9355	0.44354
D1	48,182,578	7,227,386,700	46,136,740	6,360,715,666	0.97493	0.93193	0.44096
D2	45,831,210	6,874,681,500	44,204,866	6,251,171,750	0.97841	0.93731	0.43161
D3	39,201,378	5,880,206,700	37,858,956	5,406,190,324	0.97767	0.9361	0.43261
D4	47,225,408	7,083,811,200	45,464,764	6,379,981,760	0.97764	0.93584	0.43229
D5	69,988,152	10,498,222,800	67,141,642	9,274,626,748	0.97757	0.9361	0.42876
D6	45,270,878	6,790,631,700	43,757,770	6,242,565,426	0.97762	0.93568	0.42883

**Table 2:** The Alignment of transcriptome sequencing to the reference genome

Sample_name	Total_reads(bp)	Unique_mapped(%)	Multiple_mapped(%)	Unmapped(%)
C1	18,733,298	88.15	4.4	7.45
C2	24,499,990	90.16	4.46	5.38
C3	20,776,358	89.95	4.48	5.57
C4	20,219,003	88.35	4.64	7.01
C5	21,674,196	87.62	4.97	7.41
C6	15,584,650	88.63	4.41	6.96
D1	23,068,370	87.8	4.55	7.65
D2	22,102,433	88.57	4.35	7.08
D3	18,929,478	88.86	4.46	6.68
D4	22,732,382	88.3	4.49	7.21
D5	33,570,821	87.08	4.88	8.04
D6	21,878,885	88.81	4.6	6.59

**Table 3:** New transcripts annotated using public databases

Item	Count	Percentage
All	62,088	100%
Annotation	33,898	55%
KEGG	9,791	16%
Pathway	7,828	13%
Nr	33,763	54%
Uniprot	33,375	54%
GO	24,928	40%
KOG	337	1%
Pfam	21,382	34%
TF	966	2%

### New transcript annotations from different databases

**Fig. 2:**
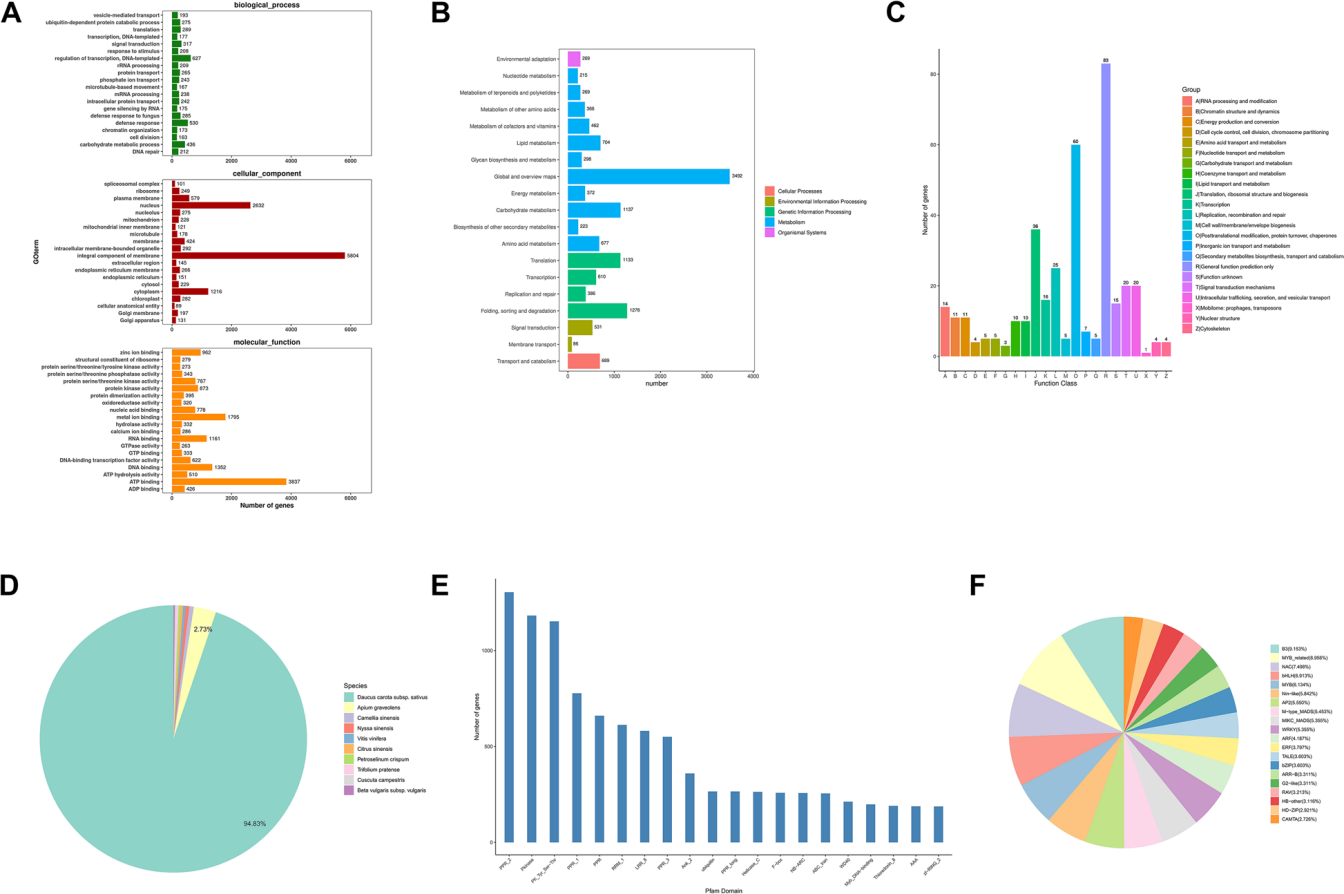
Database annotation of new transcripts based on sequence similarity aignment. **A** Gene Ontology taxonomic annotations. **B **KEGG metabolic pathway classification annotation. **C** KOG classification annotation. **D** Nr annotation species distribution. **E** pfam conserved domain annotation. **F** Taxonomic annotation of transcription factors

 The functions of all transcripts were initially annotated using the Gene Ontology. In terms of biological processes, there are numerous genes involved in the regulation of transcription, defense response, and carbohydrate metabolic processes. In terms of cellular components, the main cellular structures or regions involved are the membrane, nucleus, and cytoplasm. In terms of molecular functions, the genes involved are mainly related to ATP-binding, mental ion binding, and RNA binding (Fig. 2A). Transcripts were annotated using the Pathway database, revealing that the number of transcripts involved in metabolism and genetic information processing was high, while the number of transcripts involved in environmental adaptation, membrane transport, etc. was minor (Fig. 2B). The results of KOG database annotation showed that there were a large number of transcripts involving general function prediction, post-transcription modification, protein turnover, and chaperones (Fig. 2C). The NR database gives the top ten species with the highest matching value. The dominant species, *Daucus carota* subsp. sativus (29,104), accounts for 94.8% of all sequences, followed by *Apium graveolens* (837) (Fig. 2D). These transcripts contain additional structural domains: pentatricopeptide repeat (PPR), protein kinase (Pkinase), RNA recognition motif (RRM), leucine-rich repeat (LRR), and ankyrin repeat (Ank) domains (Fig. 2E). The results of transcription factor annotation revealed that these transcripts contain more transcription factor families, including B3, MYB_related, NAC, bHLH, MYB, Nin-like, and AP2 (Fig. 2F).

### Alternative splicing and gene structure analysis of novel transcripts

**Fig. 3:**
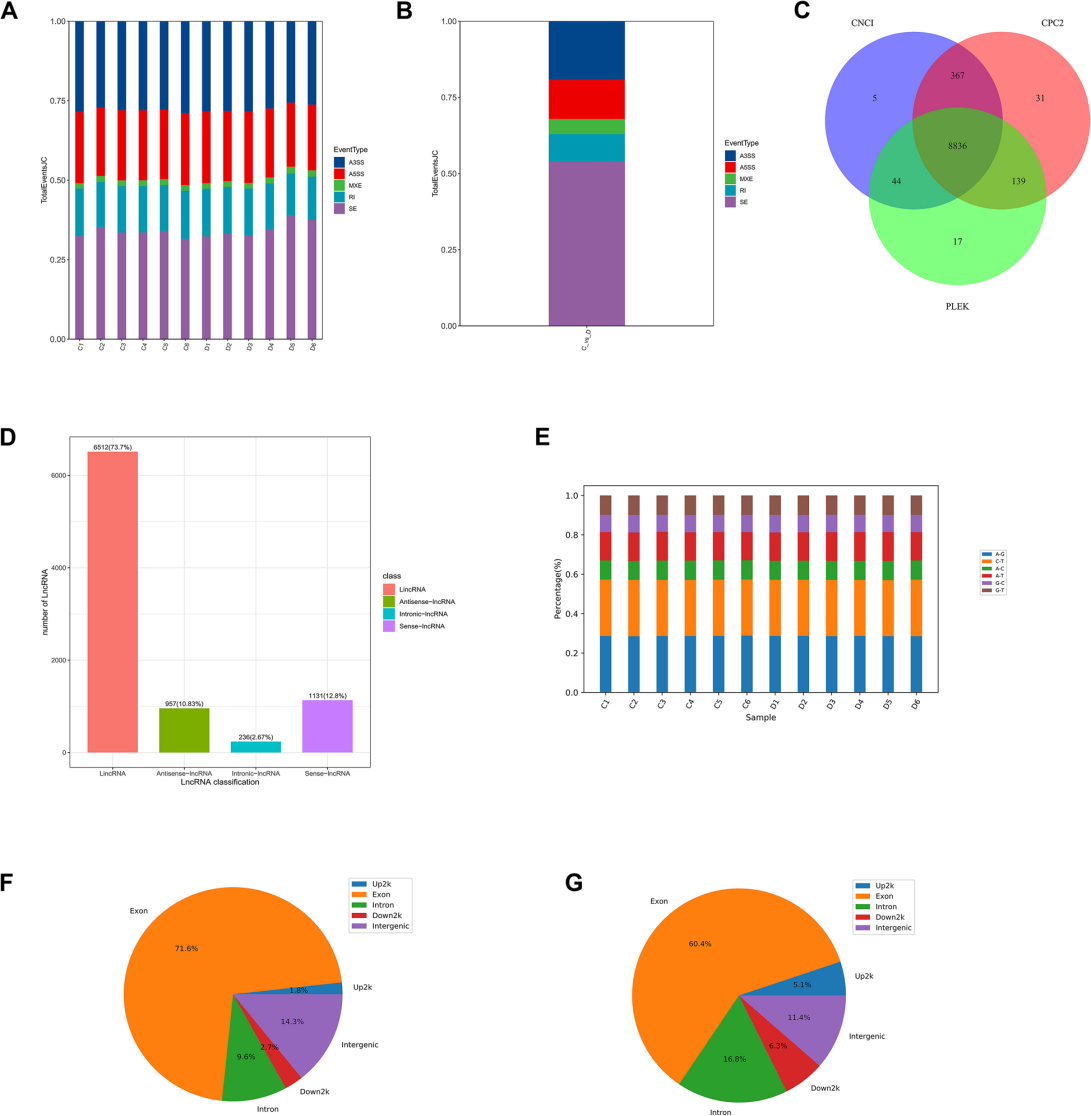
Gene structure and alternative splicing analysis. **A** Type distribution of alternative splicing events in all samples. A3SS is an alternative 3’ splice site, A5SS is an alternative 5’ splice site, MXE is mutually exclusive exons, RI is a retained intron, and SE is a skipped exon. **B** The distribution of differently variable splicing. **C** LncRNA transcript quantity prediction based on three methods: CPC2, CNCI and PLEK. **D** Classification of all lncRNAs. **E** SNP types in all samples. **F** Regional distribution of SNP loci. **G** Regional distribution of In/Del loci

 Prior to alternative splicing analysis, the length of transcript was optimized by comparing it to known transcripts across the genome. The results showed that the alternative splicing types of these samples included alternative 3’ splice site (A3SS), alternative 5’ splice site (A5SS), MXE (mutually exclusive exons), RI (retained intron), and skipped exon (SE). The SE type of transcripts accounted for the most, followed by A3SS and A5SS (Fig. 3A). Comparison of differential alternative splicing events between groups C and D also showed similar results (Fig. 3B). Three methods were used to predict the number of noncoding transcripts, showing that CPC2, CNCI, and PLEK can jointly predict 8,836 lncRNAs, and CPC2, CNCI, and PLEK can independently predict 31, 17, and 5 lncRNAs, respectively (Fig. 3C). All of the lncRNAs found can be classified into four groups. The sequence numbers of lincRNA, sense-lncRNA, antisense-lncRNA, and intronic-lncRNA are 6,512, 1,131, 957, and 236, respectively (Fig. 3D). To obtain the SNPs and In/Del sites of these transcripts, we performed variant detection on transcriptome data. The results showed that the SNP types in all transcripts mainly had six base substitutions, with the conversion ratio of A-G and C-T being higher than those of A-C and G-C (Fig. 3E). In addition, the distribution of these SNP sites also has certain rules. Nearly 71.6% of the SNPs are located in the exon region, 14.3% are located in the intergenic region, and a few are located in the promoter region (Fig. 3F). We also analyzed insert and delete sites in transcripts, and the results showed that In/Del sites in exons were the most prevalent, (60.4%), followed by introns and intergenic regions (16.8% and 11.4%, respectively) (Fig. 3G).

### Quantification of genes and sample correlation analysis

**Fig. 4:**
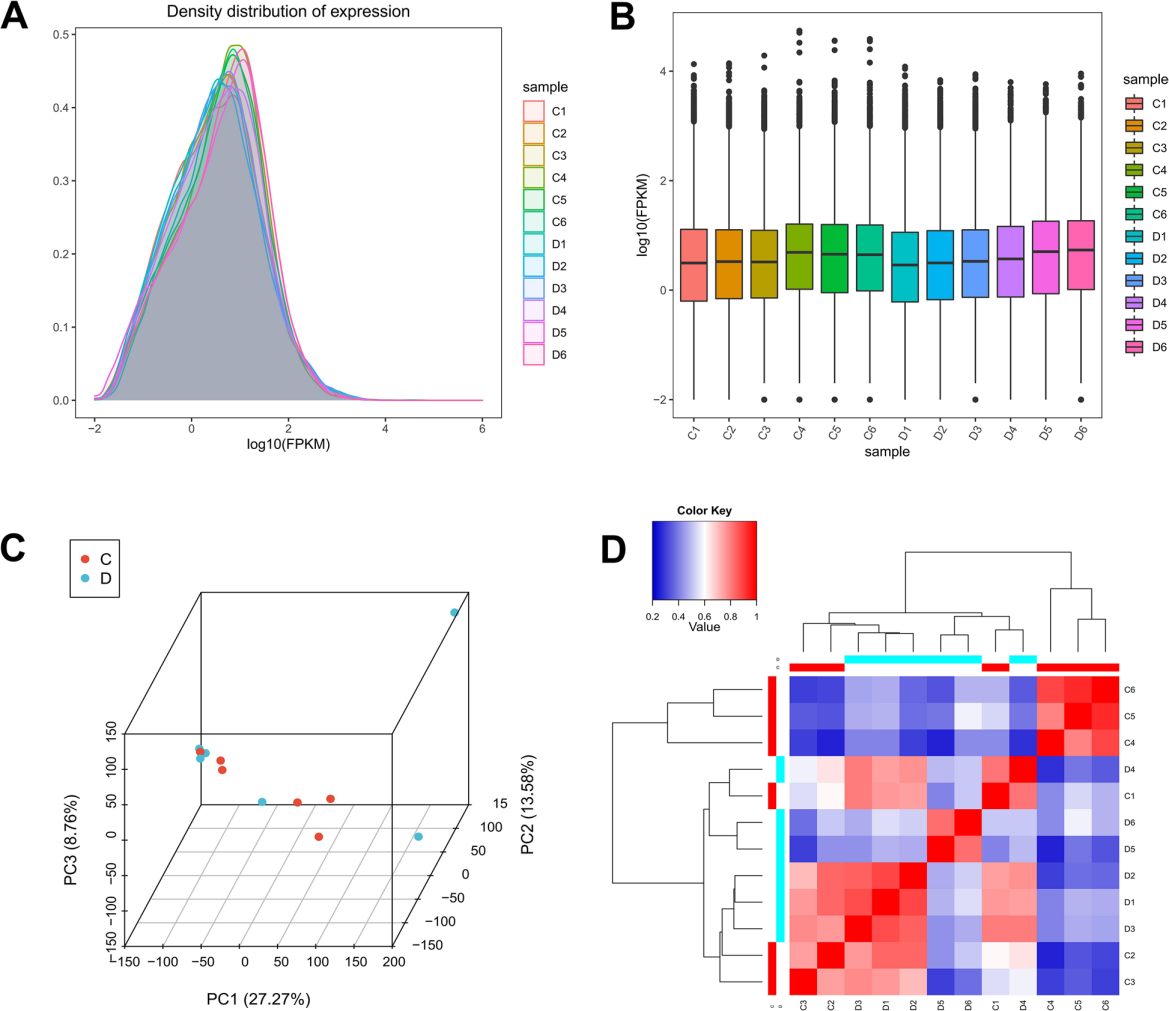
Gene quantification and sample correlation analysis. **A** Gene expression density distribution of all samples. The x-axis is the logarithm of the expression level (FPKM value) taking base 10, and the y-axis is the density of genes with different expression levels. **B** Comparison of the degree of dispersion of gene expression levels in different samples. From top to bottom are the maximum value, upper quartile, median, lower quartile, and minimum value. **C** Principal component analysis of all samples. **D** Correlation matrix analysis of all samples

 The convertion of the counts to FPKM values allowed for the quantification and evaluation of the copy number of all transcripts and genes. The density distribution of gene expression shows that the overall pattern of gene expression for each group of samples is unusual, with expression level ranging from − 2 to 2 (Fig. 4A). To show the degree of dispersion of gene expression levels between different samples, the boxplot was used to illustrate the overall levels of gene expression. The results showed that the expression levels of these samples were consistently maintained at the same level. The expression levels in the C3, C4, C5, C6, D3, and D6 groups had a high degree of dispersion (Fig. 4B). Principal component analysis showed that the total contribution rate of the first three principal components (PC1, PC2, and PC3) accounted for 49.61% of the total variance. However, some samples were not completely distinguished (Fig. 4C). Through the correlation analysis of the two groups of samples, it was shown that there exist substantial differences between the two groups. D1, D2, and D3 have a higher correlation with C2 and C3. D5 and D6 have a higher correlation. The connection between C4, C5, and C6 is comparatively higher. However, C1 and D4 did cluster into one branch, which may be caused by inconsistent sampling (Fig. 4D).

### Functional analysis of DEGs and protein-protein interaction network

**Fig. 5:**
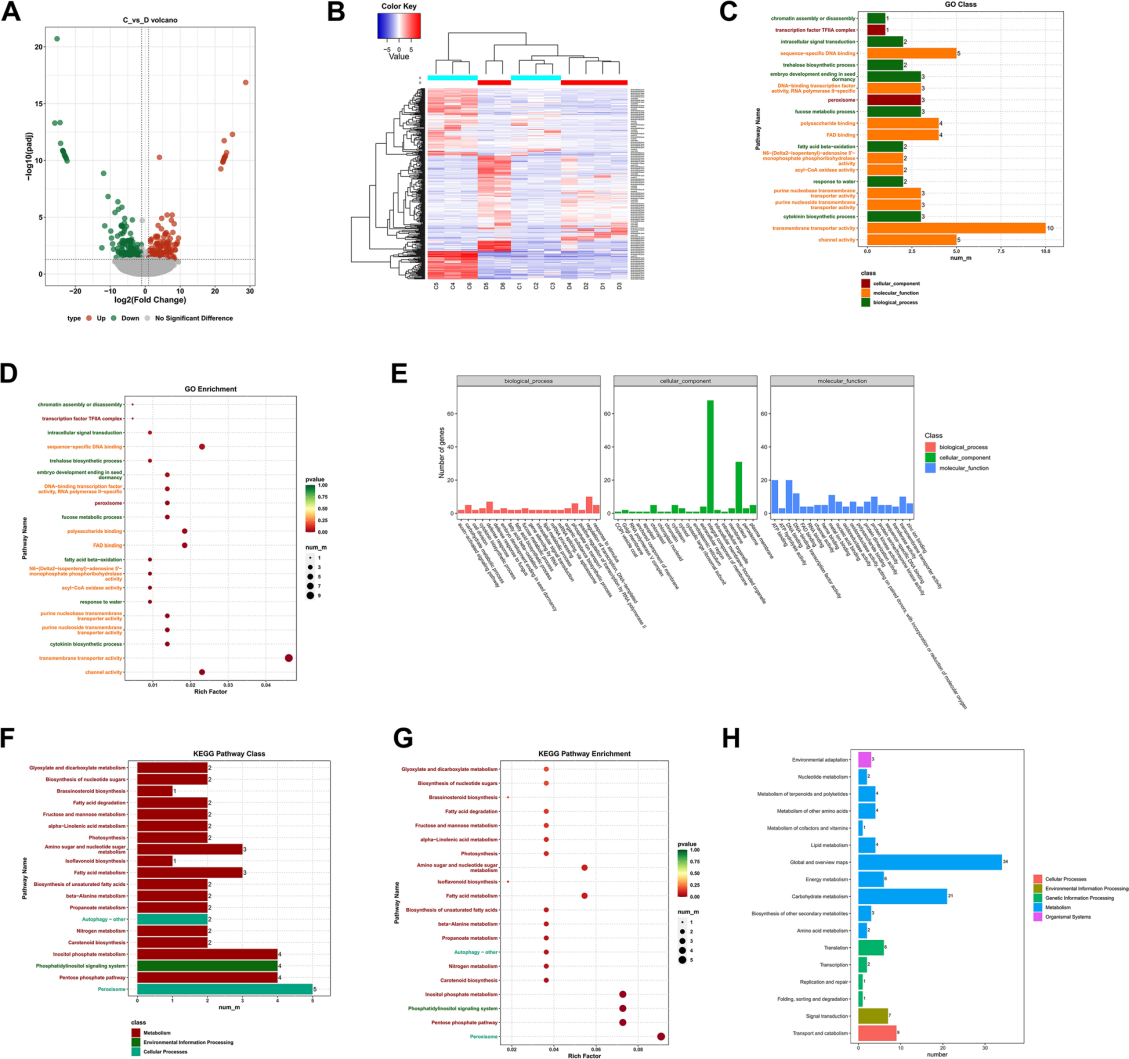
Analysis of differentially expressed genes in two groups of samples. **A** Volcano map of differentially expressed genes. The abscissa is the base 2 logarithmic value of the expression difference of a certain gene in two samples, and the ordinate is the base 10 logarithm of the false discovery rate. **B** Cluster analysis of differentially expressed genes. **C** Barplot diagram of GO enrichment analysis of differentially expressed genes. **D** Dotplot diagram of GO enrichment analysis of differentially expressed genes. **E** GO term functional classification of differentially expressed genes. **F** KEGG pathway enrichment analysis barplot diagram of differentially expressed genes. **G** KEGG pathway enrichment analysis dotplot diagram of differentially expressed genes. **H** KEGG pathway functional classification of differentially expressed genes

 DESeq2 software was used to identify differentially expressed genes based on the expression level of genes in each sample. A total of 327 significantly different genes were screened from C and D groups, including 164 up-regulated genes and 163 down-regulated genes. Volcano plots visually represent differentially expressed genes (DEGs) that exhibit significant up- or down-regulation (Fig. 5A). The hierarchical clustering analysis of DEGs in samples showed distinct expression patterns across the C and D groups. Notably, the C4, C5, and C6 groups had the highest expression levels among the samples, while the D5 and D6 groups also demonstrated elevated gene expression. However, the expression levels of these differentially expressed genes in other samples were found to be modest (Fig. 5B). GO classification and enrichment analysis showed that these DEGs include transmembrane transporter activity, channel activity, sequence-specific DNA binding, polysaccharide binding, and other functions (Fig. 5C and D). The functional classification of GO terms shows that these differentially expressed genes mainly appear in integral component of the membrane, the nucleus. Molecular functions involve DNA-binding, ATP-binding, and transmembrane transporter activity. Biological processes mainly involve regulation of transcription, defense response, and response to stimulus( Fig. 5E). The classification and enrichment analysis of KEGG pathway showed that genes involved in the peroxisome, hexose phosphate pathway, phosphatidylinositol signaling system, inositol phosphate metabolism, fatty acid metabolism, and other pathways were greatly enriched (Fig. 5F and G). Furthermore, a greater number of genes were found to be implicated in carbohydrate metabolism, energy metabolism, signal transduction, transport, and catabolism (Fig. 5H). The differential protein interaction network was constructed by leveraging pre-existing interaction models sourced from the STRING protein interaction library, including protein sequence alignments with reference species. This approach facilitated the comparison of transcriptomes. The results showed that a total of 1,457 pairs of proteins had interactions with each other. Among them, BHQH00038784 and BHQH00018093 have the strongest interactions. These interactions corresponded to AT3G54050.1 (high cyclic electron flow 1) and AT3G60750.1 (transketolase). BHQH00025576 and BHQH00014113 corresponded to AT1G35910.1 (trehalose-6-phosphate phosphatase D) and AT1G78580.1 (trehalose-6-phosphate synthase). Furthermore, it is worth noting that BHQH00030012 has the highest number of interactions with other differential proteins, corresponding to AT2G39800.1 (pyrroline-5-carboxylate synthase 1). BHQH00001954 corresponded to AT5G13650.2 (elongation factor family protein).

### Expression profiling analysis of DEGs

 We analyzed several important gene families involved in coumarin biosynthesis. A total of 19 UbiA candidate genes were identified, and this family of genes is associated to the prenylation of the coumarin. Among them, *BHQH00008828*, *BHQH00026491*, *BHQH00015746*, *BHQH00011521, BHQH00008821*, and *BHQH00012597* were highly expressed during bolting phase (Fig. 7A). However, *BHQH00008834*, *BHQH00005059*, and *BHQH00043429* were hardly expressed. The *UGT* family primarily participates in the post-glycosylation of UDP-glucose in coumarin compounds. A total of 31 *UGT* candidate genes were identified but only *novel21186*, *novel17272*, and *BHQH00040675* exhibited high expression. Many other UGTs showed low expression levels (Fig. 7B). In addition, we also screened out 50 *OMT* candidate genes, which are related to the *O*-methylation modification in the phenylpropane biosynthetic pathway. The results indicated that the expression level of *BHQH00013672*, *BHQH00038285*, *BHQH00027437*, and *BHQH00025634* was higher, while some *OMT* genes decreased after bolting, like *novel21357* (Fig. 7C). The peroxidases (*PRX*) family is involved in lignin polymerization. A total of 100 possible PRX candidates were discovered, and over two-thirds of these PRXs exhibited little or no expression (Fig. 7D). *BHQH00039750*, *BHQH00009095*, and *BHQH00017842* showed high expression levels, several genes, such as *BHQH00015002*, showed high levels of expression after bolting. On the basis of the KEGG pathway enrichment analysis, some *ATGs* with varied functions were identified (Fig. 5). We further analyzed the ATGs that could cause root senescence and identified 73 ATG candidates. The expression levels of *BHQH00024954*, *BHQH00032766*, *BHQH00003585*, *BHQH00012531*, *BHQH00044372*, and *BHQH00030339* were higher during bolting. *BHQH00009837*, *BHQH00013830*, and *novel8944* were highly expressed after bolting (Figure S1).

## Discussion

### Identification of novel transcripts, functional annotation and gene structure analysis

A total of 62,088 novel transcripts were identified by alignment with the reference genome. Among these transcripts, nearly half are unknown and over 26,000 are multiexon structures. This indicates that the majority of genes exist as multiple copies of homologs, revealing the diversity and complexity of gene composition (Fig. 1). Functional annotation results showed that the biological functions of these new transcripts are mainly involved in DNA transcription and replication, protein synthesis, folding and transport, and energy metabolism. The number of PPR, Pkinase, RRM, and LRR gene families is significant when comparing conserved domains. The abundance of transcription factors, including B3, MYB, NAC, bHLH, and AP2 is considerable (Fig. 2). These results indicate that numerous genes in *P. praeruptorum* are involved in biological processes such as growth and development, signal transduction, environmental stress, and secondary metabolism [6]. In addition, we also detected alternative splicing events in these new transcripts, and different splicing patterns may be closely associated with the formation of truncated genes and the novel functions. Even though all transcripts contained five alternative splicing forms, SE, A3SS, and A5SS remained the predominant splicing variants (Fig. 3). Alternative splicing is present in almost all higher plant genes, which leads to polymorphisms in the structure and function of transcripts and proteins. *P. praeruptorum* produce alternative splicing isoforms in response to the adaptability of specific environments [19]. In addition to alternative splicing, the ratio and type of lncRNAs were also analyzed. More than 9,000 noncoding transcripts were identified, including 8,836 lncRNAs, of which long intergenic noncoding RNA (lincRNA) comprised the largest proportion at 6,512 (Fig. 3). The detection of soybean long non-coding RNA revealed that lincRNA encoded shorter transcripts and lower expression levels than other lncRNAs, but sample-specific expression was greater. These lincRNAs are involved in the development, stress response, and signal transduction of soybean. The spatiotemporal expression of the centromere region was also observed in some active cell divisions, hence indicating the involvement of lincRNA in the process of cell division [20].

### Functional annotation and expression profile analysis of DEGs

**Fig. 6:**
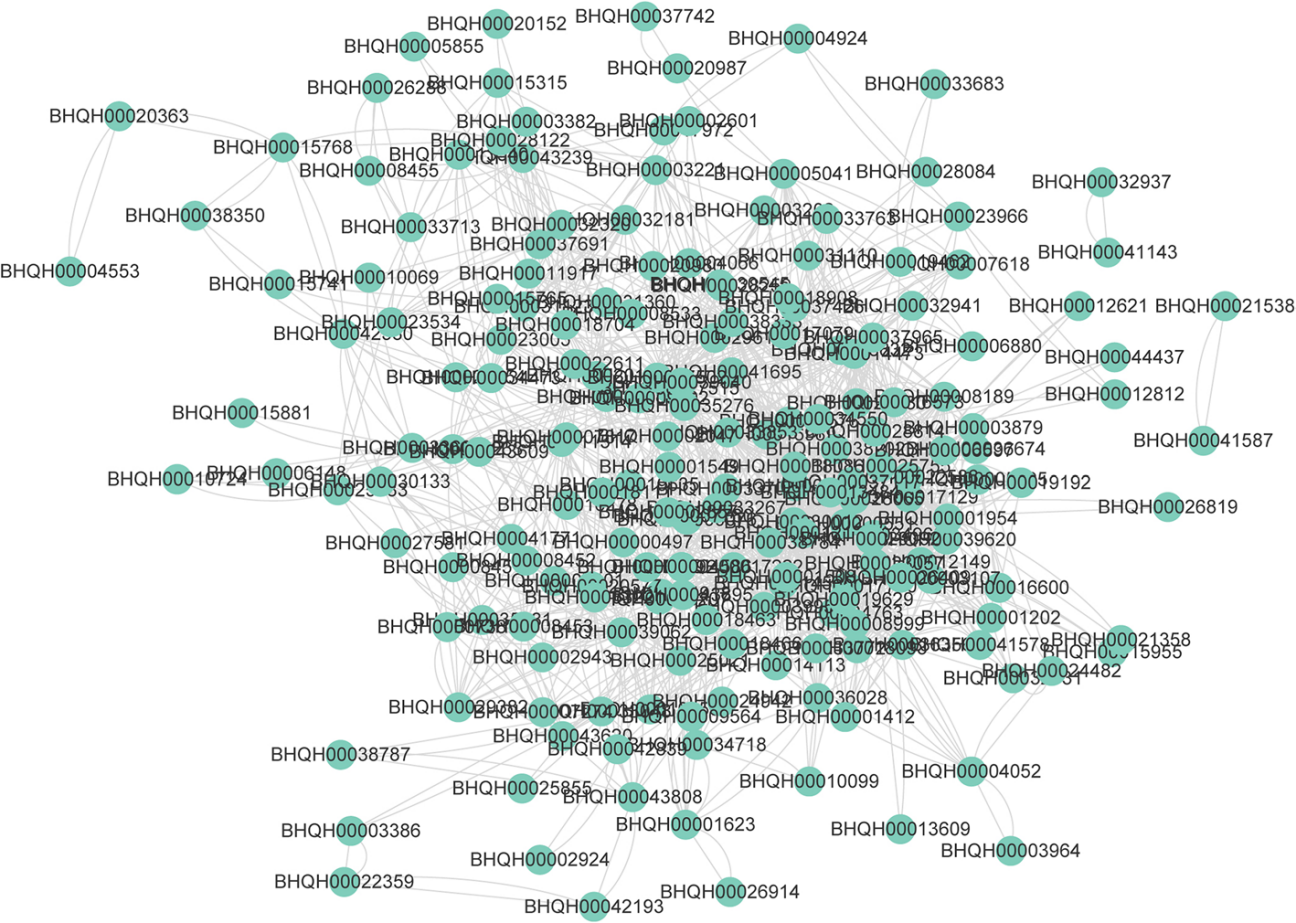
Differential protein-protein interaction network analysis

 Quantitative analysis of transcripts and genes showed that most genes had low or no expression, while only a limit subset of genes demonstrated strong expression in both sample groups. The distribution of expression levels in all samples exhibited a low degree of discreteness (Fig. 4). Principal component analysis and correlation analysis show that the two groups of samples are not completely separated, like the C1, C2, C3, and D1 groups. The heat map reveals a division of the 12 samples into two distinct branches. Specifically, C4, C5, and C6 are in one branch, while the remaining samples were further divided into three small branches. This may be related to differences in sampling. To elucidate the specific impact on gene expression after bolting, we conducted a screening of potential genes associated with root lignification and coumarin biosynthesis. This screening was performed on a pool of 327 genes that exhibited significant differences. Subsequently, we created a protein interaction network to analyze the differential effects (Figs. 5 and 6). Our previous studies had shown that most of the carbon and nitrogen sources in the roots would gradually transfer to the apical tissues for reproductive growth after bolting [13]. The expression of numerous crucial enzyme genes implicated in the biosynthesis of coumarin, including *PAL*, *C4H*, *HCT*, *COMT*, and *CCoAOMT*, exhibited a decrease, but some ABC transporters involved in transmembrane transport demonstrated an up-regulation [5]. Genes involved in transmembrane transport, ion channels, cytokinin synthesis, hexose phosphate metabolism, inositol phosphate metabolism, DNA-binding, phosphatidylinositol signaling, fatty acid metabolism, the peroxisome, and autophagy were significantly enriched in this study. These results indicated that many genes associated with material and energy metabolism were triggered after bolting [6].

**Fig. 7:**
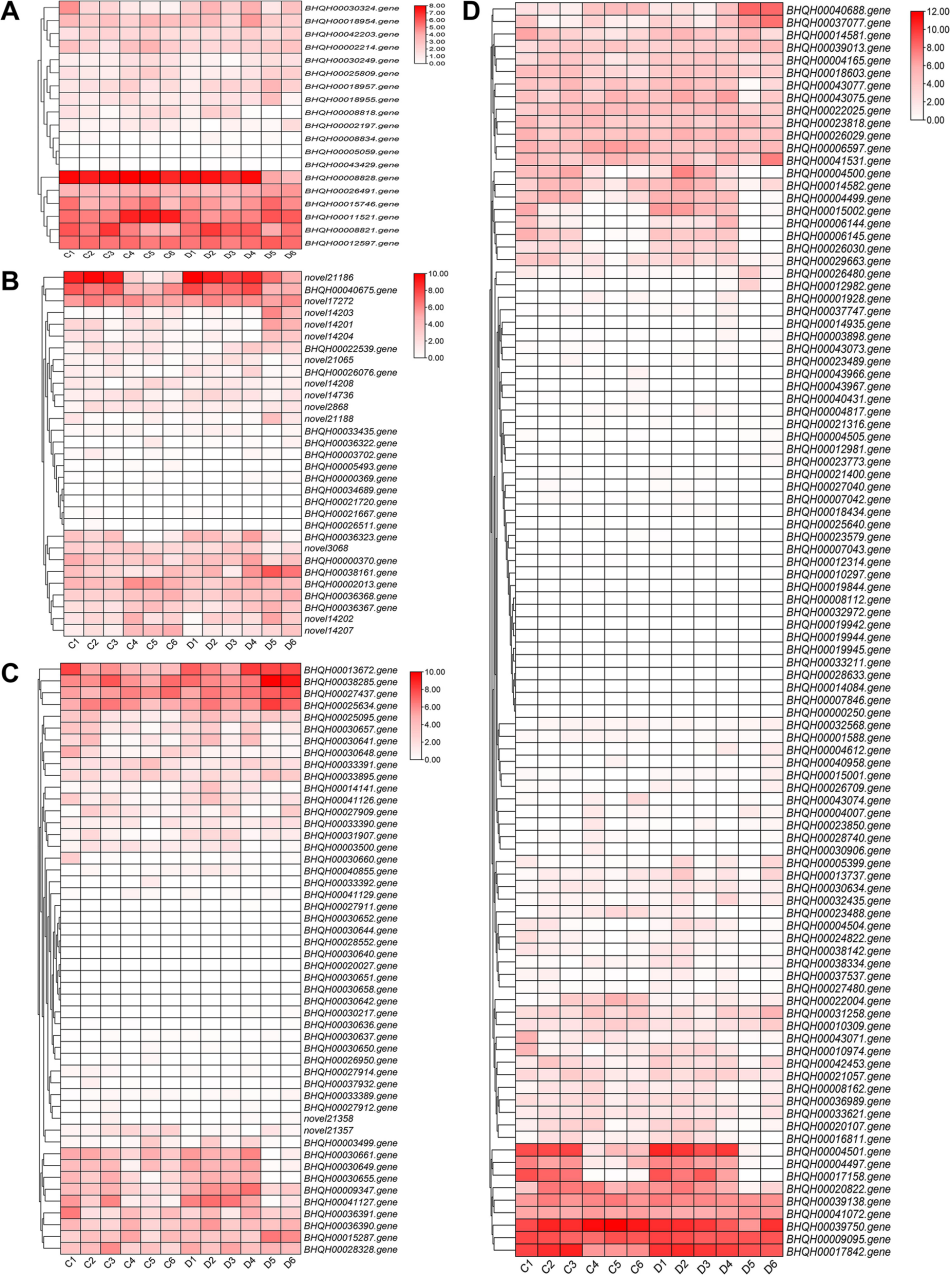
Expression profiles of differentially expressed genes before and after bolting. **A** UbiA family genes. **B** UGT family genes. **C** OMT family genes. **D** PRX family genes

Moreover, the expression patterns of *UbiA*, *UGT*, and *OMT* genes involved in coumarin biosynthesis were studied. Studies have shown that one branch of UbiA is responsible for the prenylation of coumarin, including O-prenyltransferase and C-prenyltransferase [21, 22]. The UbiA family catalyzes the conjugation of substrates to isopentenyl monomers to form linear or cyclic products. Among them, the prenyltransferase (PT) with the umbelliferone as the substrate can form diverse coumarin compounds with different structures, and this kind of ingredient is also the main active ingredient of *P. praeruptorum*. A total of 19 *UbiA* candidates were identified, and *BHQH00030324*, *BHQH00008821*, *BHQH00008818*, and *BHQH00008828* may be involved in the prenylation of coumarins. The expression levels of *BHQH00008828* and *BHQH00008821* were high before and after bolting, while the expression level of *BHQH00030324* decreased after bolting (Fig. 7). The UGT family is widely spread in higher plants and participates in the glycosylation modification of active substances to facilitate the transmembrane and transport of small molecules [23]. Glycosylation modification plays an important role in the transmembrane transport and activity of plant secondary metabolites. Coumarin is glycosylated to form a series of isomers with different stereoselectivities when the furan- and pyranocoumarin skeletons are formed. A total of 31 *UGT* genes were identified, but only a few were highly expressed during bolting. OMT genes are a large number of post-modification enzymes related to O-methylation. Many processes in the lignin, coumarin, and flavonoid biosynthesis are inseparable from OMT genes. A similar situation also appeared in the *OMT* family. Among the 50 *OMT* genes identified, only four were highly expressed, indicating that most genes are the outcome of large-scale gene duplication and are redundant.. These genes may be involved in the methylation modification of phenylpropanoids during the bolting [24]. Our previous studies showed that the expression of apoptosis-related genes and peroxidases may be related to secondary wall thickening and root lignification after bolting [5]. At this time, part of the coumarin is degraded and part is transferred to the ground for the growth of vegetative organs. Some *PRX* genes in peroxisomes were identified. Expression profile analysis showed that nine *PRX* genes, including *BHQH00017842*, were highly expressed during the bolting process, while more than two-thirds of *PRX* genes were lowly or not expressed. The mechanism by which Class II PRXs are involved in lignin polymerization remains uncover. In addition, we identified autophagy-related genes (ATGs) for the first time in the genome of *P. praeruptorum.* Autophagy is a protein degradation pathway that relies on lysosomes and vacuoles. Autophagosomes act as garbage removal stations, and inactivation of autophagy leads to the accumulation of intracellular proteins. A total of 73 ATG family genes were screened. Nearly two-thirds of the genes were up-regulated during bolting, which may be related to the processes of apoptosis and cargo transport upon lignification. In the future, when studying the quality improvement of *Peucedanum spp.*, in addition to focusing on the self-regulation of coumarin biosynthetic genes, much attention should be paid to the negative regulation of delaying lignification and the functional characterization of protein transport and autophagy.

## Conclusions

Many plants in the Apiaceae family have unique odors and ornamental properties and are also broadly used in food flavoring and clinical medicine. *P. praeruptorum* is also an important traditional Chinese medicine that has been utilized in China for centuries. However, after bolting, the roots of *P. praeruptorum* are gradually lignified, resulting in a sharp drop in the content of coumarin components. Consequently, this reduction in yield renders the plant unsuitable for medicinal purposes. New essential genes were identified from the comparative transcriptome of the bolting stage by the combined examination of the genome and transcriptome. Over 60,000 new transcripts were identified through genome comparison, of which more than 30,000 were novel genes. Through database annotation and functional enrichment analysis, it was indicated that transmembrane transporters, channel proteins, DNA-binding proteins, polysaccharide-binding proteins, etc. were enriched in large quantities. The functional enrichment of KEGG pathway showed that the genes related to the peroxisome, hexose phosphate pathway, phosphatidylinositol signaling system, and inositol phosphate metabolism pathway were greatly enriched. Some *UbiA*, *UGT*, and *OMT* family genes were abundantly expressed during bolting stage. Only a small number of *PRX* genes associated with lignin polymerization were highly expressed. Additionally, a total of 73 *ATGs* were identified from the functionally enriched pathways. The expression profiling showed that nearly one-third of *ATG* genes displayed high levels of expressed during the bolting stage. These results may provide scientific ideas for the identification of functional genes in *P. praeruptorum*.

### Methods

#### Sample collection, data collection, and genome comparison

The material of *P. praeruptorum* used in this experiment was collected from Donghekou Town, Lu’an, Anhui Province. The geographical coordinates are 116.6567◦ east longitude and 31.4044◦ north latitude. The samples were collected from March to November of that year, approximately every 15 to 30 days. The samples for the analysis were taken 15 days after bolting. The base plant was identified by Professor Bangxing Han of West Anhui University as the Peucedanum genus in the Apiaceae family. The voucher specimen deposited at the herbarium of West Anhui University (Luan, China) is publicly available and the deposition number is 120,951–201,706. After the samples were cleaned and covered with soil, they were dried with absorbent paper, placed in liquid nitrogen for quick freezing, and then the total RNA was extracted and a cDNA library was constructed for subsequent NGS. The remaining samples were stored in a refrigerator at -80 °C .

The transcripts of six samples were sequenced and assembled using the Illumina Hiseq platform [5, 13]. By removing adapter sequences and low-quality sequences, the data information of all samples was obtained after quality control [25]. The original image data files obtained by high-throughput sequencing were transformed into original unfiltered reads through base calling analysis and converted into FASTQ files. To assure the reliability of the information analysis results, it is necessary to apply filtration to the raw sequencing data, which are still stored in the FASTQ format. To obtain accurate data for subsequent analysis, fastp (v. 0.21.0) software was used to filter the original fastq data. Fastqc (v. 0.11.9) was used for quality control of the filtered data. The original data has been uploaded to the NCBI SRA database; the accession number of the BioProject was PRJNA714368. The filtered transcriptome sequence was compared with the reference gene using Star (v. 2.7.9a) software [26]. The randomness of reads on the reference gene was evaluated., The of randomness of mRNA fragmentation can be determined by sequencing the position distribution of reads on the gene. IGV was used to visualize the alignment results of sequenced Reads and reference genome sequence files, species reference genome sequences, and annotation files.

### Prediction and functional annotation of novel transcripts

StringTie (v.2.1.4) software was used for transcript assembly [27]. To consolidate the transcript gtf files obtained from individual samples, the merge function of StringTie can be employed. This will facilitate the merging of gtf files produced from all samples into a single file. The gffcompare (v. 0.12.1) was employed to do a comparative analysis between the merged transcripts and the known transcripts of the genome. This analysis aimed to identify novel transcripts and genes, as well as enhance the current annotations. TransDecoder (v. 5.5.0) was used to predict the coding sequence and CDS length of the newly identified transcripts. Functional annotation of transcripts was performed using seven databases: Nr, Pfam, Unipro, KEGG, GO, KOG/COG, and PATHWAY. The protein sequence encoded by the transcript was compared with Uniprot, Nr, and KEGG for diamond blastp (v. 2.0.6.144) to obtain the functional information of the sequence and the metabolic pathway information involved [28]. KEGG annotation was performed using KOBAS (v. 3.0), KEGG ORTHOLOGY and PATHWAY [29, 30]. Based on the relationship between the databases, the KOG/COG annotations and the classification were performed [31]. Another method was the motif similarity search. Hmmscan (v. 3.3.2) was used for domain prediction to obtain conserved sequences, motifs, structural domains, etc.

### Gene expression quantification and DEG analysis

RSEM was used to get the number of reads aligned to each transcript for each sample. These reads are first converted to FPKM to obtain the expression levels of genes and transcripts [32]. A Pearson analysis was performed on the correlation between the two samples. The principal component analysis shared the similarity between samples. Differential expression analysis of gene expression was performed using DESeq2 (v.1.26.0) software [33]. The screening threshold for DEGs was padj < 0.05 and |log_2_FoldChange| > 1. The FDR value was used to filter out significantly different genes. ClusterProfiler (v. 3.14.3) was used to perform GO and KEGG pathway enrichment analyses of DEGs. The value range of p.adjust (qvalue) is between 0 and 1. The relationships between proteins in the STRING protein interaction database were used to check how different genes interact with each other [34]. Since the gene set of *P. praeruptorum* is not in the database, the gene sequence was first transformed into a protein sequence. Then, the protein sequence of *A. thaliana* in the string database was compared to the protein sequence of *P. praeruptorum* to make an interaction network. The protein-protein interaction network was visualized using Cytoscape (v. 3.10.0) software.

### Gene structure and variation analysis

First, structure optimization was performed on the original annotated transcripts. The gffcompare (v. 0.12.1) software was used to conduct a comparative analysis between newly identified transcripts and previously annotated transcripts throughout the genome. If there is a region of the transcript outside the boundary of the original transcript, the upstream and downstream of UTR will be extended, therefore rectifying transcript boundaries. rMATS (v.4.1.1) software was used to analyze The type of alternative splicing present in each sample [35]. Furthermore, rMATS software was also used to detect the difference in alternative splicing between samples. Transcription factor was predicated using PlantTFDB 5.0 software. The coding potential of the newly identified transcripts was predicted using CNCI (v. 2.0), CPC2 (v. 1.0.1), and PLEK [36]. The samtools (v. 1.11) software was used to sort the bam files [37]. GATK (v. 4.2.0.0) was used to detect the variation of the transcriptome data and obtain high-quality SNP and In/Del [38].

### Expression profiles of key genes before and after bolting

The expression patterns of key genes involved in the coumarin biosynthesis pathway, lignin polymerization, and autophagy-related pathways were examined. First, the hidden Markov models of the UbiA (PF01040), OMT (PF01596 and PF00891), UGT (PF00201), and PRX (PF00141) families were constructed using the HMMER program. For autophagy-related proteins, they are divided into ATG1 (PF07714), ATG2 (PF12624), ATG3 (PF03987), ATG4 (PF03416), and ATG5 (PF04106) based on the existing Arabidopsis nomenclature and conserved domains: ATG6 (PF04111), ATG7 (PF16420), ATG8 (PF02991), ATG9 (PF04109), ATG10 (PF03987), ATG11 (PF10377), ATG12 (PF04110), ATG13 (PF10033), ATG16 (PF10033), and ATG101 (PF10033) A total of 15 subgroups. The expression levels were normalized by log_2_ (FPKM) for the construction of expression profiles.

### Supplementary Information

**Additional file 1:Figure S1.** Expression profile analysis of ATG family genes before and after bolting. **Table S1.** KO-enriched terms and genes in the two groups of samples before and after bolting. **Table S2.** GO functional classification terms and genes of two groups of samples before and after bolting. **Table S3.** Differentially expressed transcription factors in two samples before and after bolting. **Table S4.** Source and target genes for differential protein interaction network construction. **Table S5.** FPKM values and annotation information of five gene families in two samples before and after bolting.

## Data Availability

The genome assembly and annotations have been deposited at GenBank database and figshare with the accession number PRJNA910498 and the available link (10.6084/m9.figshare.21743984.v1). The transcriptome data were deposited at the GenBank SRA database with accession number PRJNA714368.

## Abbreviations

PPR: Pentatricopeptide repeat
Pkinase: Protein kinase
RRM: RNA recognition motif
LRR: Leucine-rich repeat
OMT: *O*-methyltransferase
UGT: UDP-glycosyltransferase
ATG: Autophagy-related gene
PT: Prenyltransferase
PRX: Peroxidase
A3SS: Alternative 3’ splice site
A5SS: Alternative 5’ splice site
MXE: Mutually exclusive exons
RI: Retained intron
SE: Skipped exon
