# Supplementary figures and images for: Genome sequencing-based transcriptomic analysis reveals novel genes in Peucedanum praeruptorum
Source: BMC Genom Data. 2023 Sep 18;24:53. doi: 10.1186/s12863-023-01157-y (PMC10506206; doi:10.1186/s12863-023-01157-y)

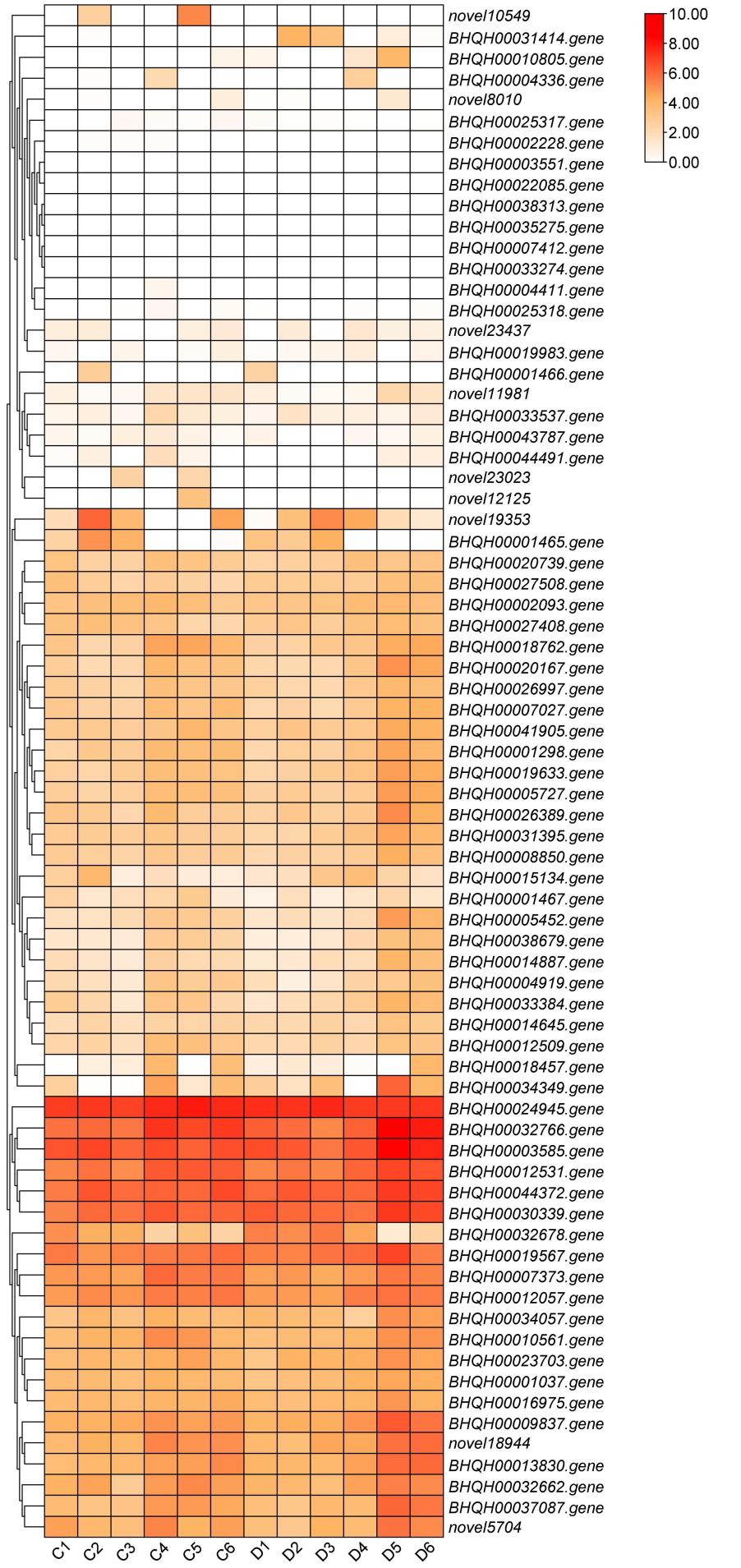


**Figure S1** Expression profile analysis of ATG family genes before and after bolting

Supplement: Supplementary file 1 — Additional file 1:Figure S1. Expression profile analysis of ATG family genes before and after bolting. Table S1. KO-enriched terms and genes in the two groups of samples before and after bolting. Table S2. GO functional classification terms and genes of two groups of samples before and after bolting. Table S3. Differentially expressed transcription factors in two samples before and after bolting. Table S4. Source and target genes for differential protein interaction network construction. Table S5. FPKM values and annotation information of five gene families in two samples before and after bolting. [file 12863_2023_1157_MOESM1_ESM.zip › Additinal file 1.docx]
